# A Methodology to Identify and Prioritize Gene Candidates for Human Disease

**DOI:** 10.3389/fgene.2012.00133

**Published:** 2012-07-18

**Authors:** Jesus Sainz

**Affiliations:** ^1^Spanish Research Council, Instituto de Biomedicina y Biotecnologia de CantabriaSantander, Spain

In a study published this month in *Frontiers in Applied Genetic Epidemiology* (Zhi et al., 2012), the authors have studied left ventricular hypertrophy (LVH), the thickening of the myocardium of the left ventricle of the heart, which is a trait that can be used as heritable predictor of cardiovascular disease, to identify genomic variants and genes that could be used as predictive markers of increased left ventricular mass. They used next generation sequencing to produce data from the whole exome of a hypertensive population and from total mRNA of a cellular model of LVH. The authors identified 31,426 genomic missense or nonsense mutations in seven African American sibling trios with high familial left ventricular mass indexed to height (LVMH). Using regression analyses, they found out that 295 of these variants, located in 265 genes, were associated significantly to LVMH after adjusting for multiple testing. They also produced total mRNA sequence data from a cellular model of LVH, hypertrophic cardiomyocytes, that was compared to the expression data from control cardiomyocytes producing a list of differentially expressed genes (using as cut off a value of *P* < 0.05 without adjusting for multiple testing). The LVH differential expression genes were compared with the list of genes with LVMH associated variants, producing 44 genes that were common to both lists. Gene Ontology analysis of the authors 44 genes list indicates a significant enrichment of genes involved in the cell cycle process (Chi test; *P* = 0.00016, adjusted for multiple testing) and overrepresentation in the cell adhesion process. Pathway analysis indicates that 2 of the 44 common genes (THBS1 and COL6A3) are part of the signaling by Platelet-derived Growth Factor (PDGF) pathway which has been implicated in tissue remodeling, being PDGF a potent stimulator of growth. Data from the Gene Reference into Function database (GeneRIF) in NCBI, indicates that five of these common genes (HLA-B, HTT, THBS1, PAPPA, and SYNE1) have been implicated in the literature with cardiovascular risk, heart disease, or heart failure in human and in mice, and polymorphisms of another (PER3) have been associated to the sympathovagal balance in cardiac control.

When the authors adjusted the *P*-values of the differential expression data for the number of tested genes, they reduced the initial list to 11 genes with differential expression and variants associated to LVMH reaching statistical significance. Pathway analysis indicates that seven of these genes are annotated and belong to pathways such as cell cycle, signaling by PDGF, or regulation of Insulin-like Growth Factor among others. GeneRIF analysis of this new gene list, produced with more stringent criteria for the expression data that reduced to 25% the number of genes to analyze, shows that among the resulting 11 genes still were included 3 out of the 6 genes implicated with heart conditions or heart control (THBS1, PAPPA, and PER3) and one from the signaling by PDGF pathway (THBS1). This further enrichment in genes involved with heart conditions or control is in agreement with the hypothesis of the authors that the differential expression data used is a good criterion to identify heart disease risk genes and, consequently, that the novel cellular model of LVH used to obtain the expression data could be useful to provide functional information of the phenotype.

The authors used another approach to select the genes by applying a candidate gene prioritization strategy to the 44 initial genes using seven different criteria which included statistical, linkage, functional, conservation, and allele frequency data among others. This resulted in five genes that satisfied at least three of the seven criteria (HLA-B, HTT, MTSS1, SLC5A12, and THBS1). Interestingly, three of them (HLA-B, HTT, and THBS1) are among the genes implicated with heart conditions, being one of them among the 11 with significantly differential expression after adjusting for the number of tests performed. This enrichment of heart condition genes supports that the seven criteria used by the authors are good predictors to identify LVH risk genes.

None of the two main technologies used in this approach, RNA sequencing and whole exon-sequencing, is novel in the search for gene candidates to cause disease. However, the combination of whole exome sequencing, gene expression data from a cell model of the trait, and mainly, the gene prioritization strategy using seven additional criteria that includes public annotations and statistical data, provides a novel strategy.

In conclusion, the methodology used, despite all the limitations described by the authors in the article, seem to provide an enrichment of genes involved with the trait of study, provide new candidates for further studies, and a strategy that could be applied to other phenotypes with the pertinent modifications.
